# Impact of Cell-Free Fetal DNA Screening on Patients’ Choice of Invasive Procedures after a Positive California Prenatal Screen Result

**DOI:** 10.3390/jcm3030849

**Published:** 2014-07-24

**Authors:** Forum T. Shah, Kathryn Steinhaus French, Kathryn E. Osann, Maureen Bocian, Marilyn C. Jones, Lauren Korty

**Affiliations:** 1 Cancer Genetics Department, Saint Joseph Hospital, Orange, CA 92868, USA; 2 Department of Pediatrics Irvine, University of California, Orange, CA 92868, USA; E-Mails: kasteinh@uci.edu (K.S.F.); kosann@uci.edu (K.E.O.); mebocian@uci.edu (M.B.); 3 San Diego and Rady Children’s Hospital, University of California, San Diego, CA 92037, USA; E-Mail: mjones@rchsd.org; 4 San Diego, Fetal Care & Genetics San Diego, University of California, CA 92037, USA; E-Mail: ladennis@ucsd.edu

**Keywords:** prenatal, cell-free fetal DNA screening, non-invasive prenatal testing

## Abstract

Until recently, maternal serum analyte levels paired with sonographic fetal nuchal translucency measurement was the most accurate prenatal screen available for Trisomies 18 and 21, (91% and 94% detection and false positive rates of 0.31% and 4.5% respectively). Women with positive California Prenatal Screening Program (CPSP) results have the option of diagnostic testing to determine definitively if the fetus has a chromosomal abnormality. Cell-free fetal (cff-) DNA screening for Trisomies 13, 18, and 21 was first offered in 2012, allowing women with positive screens to choose additional screening before diagnostic testing. Cff-DNA sensitivity rates are as high as 99.9% and 99.1%, with false positive rates of 0.4% and 0.1%, for Trisomies 18 and 21, respectively. A retrospective chart review was performed in 2012 on 500 CPSP referrals at the University of California, San Diego Thornton Hospital. Data were collected prior to and after the introduction of cff-DNA. There was a significant increase in the number of participants who chose to pursue additional testing and a decrease in the number of invasive procedures performed after cff-DNA screening was available. We conclude that as fetal aneuploidy screening improves, the number of invasive procedures will continue to decrease.

## 1. Introduction

### 1.1. Basis and History of Prenatal Screening

**Figure 1:**
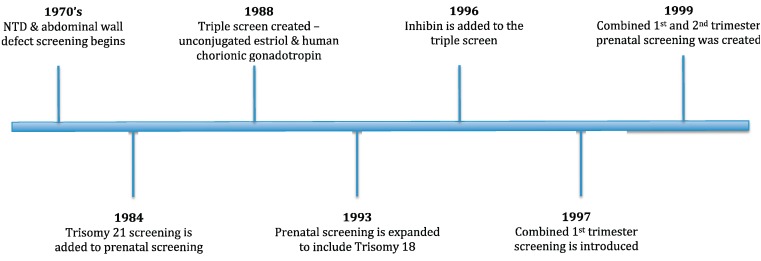
History of prenatal screening tests [7,8,9,10].

Since the introduction of second trimester screening designed to detect open neural tube defects (NTD) and ventral wall defects (VWD) in the fetus, screening programs have evolved and grown to include multiple genetic conditions, including Trisomy 18, Trisomy 21, and Smith-Lemli Opitz syndrome (SLOS) as well as NTD and VWD [1,2,3,4]. First trimester screening was introduced when it was suggested that the combination of free β-hCG and PAPP-A levels with a fetal nuchal translucency sonographic measurement and maternal age were effective screening tools [5]. Soon after, Wald proposed a new approach that integrated both first and second trimester screening methods [6] and provided better detection rates than using either method individually. A timeline for the history of prenatal screening tests can be seen in Figure 1.

Prior to the introduction of prenatal screening, women only had the option of invasive testing (*i.e.*, amniocentesis and chorionic villus sampling (CVS)) to determine if their fetuses were affected [11]. Both invasive procedures have an accuracy of nearly 100% [11,12,13]. However, each procedure has a small risk of miscarriage. In experienced hands, the midtrimester amniocentesis miscarriage risk is 1 in 400 or less [14]. The reported pregnancy risks associated with CVS are varied. According to Rhoads G. G., Jackson L. G., Schlesselman S. E. *et al.* [15], when adjusting for gestational age and maternal age, the pregnancy-associated risks of CVS are similar to that of amniocentesis. However, other researchers report an increased risk for CVS above that of mid-trimester amniocentesis [16]. 

### 1.2. California Prenatal Screening Program

The California Prenatal Screening Program (CPSP) began in 1986 and is available to all pregnant women in California [17]. It is one of the largest programs in the world, with over 400,000 pregnant women per year utilizing screening methods to determine whether a pregnancy is at risk for certain disorders (Trisomies 21 and 18, NTD, VWD, and SLOS) [17,18]. The CPSP employs fetal nuchal translucency (NT) measurement and quantification of maternal serum analytes. During the first trimester, free β-hCG and PAPP-A and during the second trimester alpha-fetoprotein, inhibin, free β-hCG and unconjugated estriol are quantified. Interpretation of maternal serum analyte levels are then adjusted based on maternal age, weight, ethnicity, smoking and diabetes status.

First Trimester combined screening consists of a blood sample (1st trimester serum) drawn between 10 weeks and 13 weeks 6 days gestation combined with a NT measurement [5,19,20]. Quadruple marker screening refers to a single blood draw (2nd trimester serum) between 15 and 20 weeks gestation [21]. A Quadruple marker blood draw can be combined with a first trimester NT measurement if first trimester serum screening did not occur (Quadruple + NT) [17]. A Serum Integrated screen combines first and second trimester serum results only, and a Sequential Integrated screen incorporates NT results with first and second trimester serum results [21]. Each screening method has different detection rates for Trisomies 18 and 21. For both Trisomies 18 and 21, sequential integrated screening has higher detection rates, and first trimester combined screening has lower detection rates (Table 1). The CPSP is not designed to screen for fetal chromosome abnormalities, apart from Trisomies 18 and 21. Previous reports have indicated that approximately 39% of karyotypic abnormalities causing phenotypic consequences may be detected by CVS and amniocentesis and do not involve Trisomies 18 or 21 [22]. Therefore, these most likely would not be detected using the CPSP.

**Table 1:** Screening tests and detection rates for trisomy 18 and trisomy 21.

	Components			Detection Rates (%) [23,24]		Highest Detection Rates (%)		Lowest False Positive Rates (%)	
Screening Category	1st T Serum	NT	2nd T Serum	T18	T21	T18	T21	T18	T21
First Trimester Combined	♦	♦		69	75				
Quadruple			♦	67	80				
Quadruple + NT		♦	♦	72	89				
Serum Integrated	♦		♦	79	85				
Sequential Integrated	♦	♦	♦	81	90				
CPSP						91	94	0.31	4.5
Cff-DNA Screening *						>99.9	99.1	0.4	0.1

Diamond indicates elements included in the screening category: 1st Trimester serum (1st T serum), nuchal translucency (NT), 2nd trimester serum (2nd T serum). California Prenatal Screening Program (CPSP) and cell-free fetal (cff). * Sequenom’s quoted highest detection rates for cff-DNA screening were utilized for this table and the study.

The CPSP has strict cut-offs for what they identify as a positive screen result. A first trimester combined screen result risk of ≥1 in 50 and a risk of ≥1 in 100 is considered positive for Trisomies 18 and 21 respectively. A screen positive result during the second trimester for Trisomy 21 is a risk ≥1 in 150 through Quadruple screening and ≥1 in 200 through Quadruple + NT, Serum Integrated, and Sequential Integrated. A second trimester screen positive result for Trisomy 18 is ≥1 in 100 through Quadruple, Quadruple + NT, Serum Integrated, and Sequential Integrated screening [23].

### 1.3. Cell-Free Fetal DNA Screening

The discovery of circulating cell-free fetal DNA in maternal plasma was the first step toward the creation of a more accurate non-invasive prenatal screen. In October 2011, Sequenom Laboratories, Inc. (San Diego, CA, USA) introduced a new clinical prenatal screen for high risk pregnancies with a greater accuracy rate for chromosomal aneuploidy than maternal serum and NT screening [25,26]. Known as cell-free fetal DNA screening, this non-invasive method can indicate if the pregnancy is at an increased risk for Trisomies 13, 18, or 21 [22]. The test detects chromosomal dosage in total circulating DNA in maternal plasma and compares it to the fractional fetal DNA concentration [27]. In Trisomies 13, 18, or 21, an increase in the dosage of each chromosome respectively, would be seen in the fetal DNA fraction in the maternal plasma [27]. Studies have shown that cell-free fetal DNA screening detection rates can be as high as 99% for both Trisomies 18 and 21 and 91.7% for Trisomy 13 [22,28,29]. Testing laboratories quote different detection rates based on their individual performance. Sequenom’s specific detection rates are 99.1% for Trisomy 21, >99.9% for Trisomy 18, and 91.7% for Trisomy 13 [30]. Sequenom’s specific false positive rates are 0.1% for Trisomy 21, 0.4% for Trisomy 18, and 0.3% for Trisomy 13 [30,31]. 

### 1.4. Diagnostic Tests vs. Screening Tests

Currently, the CPSP gives couples risk estimates for Trisomy 18 and 21 based on analyte levels in maternal serum combined with maternal age and, in the first trimester, nuchal translucency measurement; however, there are overall false positive rates of 0.31% for Trisomy 18 and 4.5% for Trisomy 21 (Table 1) [23]. In order to definitively identify an affected pregnancy, invasive prenatal testing would need to be utilized. This may not be acceptable to some couples as they may consider the risk of miscarriage to be too high. Also, women may be concerned about possible discomfort during an invasive procedure. Having the option of a highly accurate prenatal screening test, such as cell-free fetal DNA, may alleviate some of the anxiety that an expectant couple experiences when told that their pregnancy is at an increased risk for Trisomy 18 or 21. Cell-free fetal DNA screening has a significantly lower false positive rate for Trisomy 21 (Table 1) [29]. In addition, cff-DNA screening reduces the false positive and/or false negative rates and, therefore, has better accuracy rates for these disorders than the CPSP.

This study focuses on women who have screened positive through the CPSP and identifies the options women chose upon receiving these positive results.

## 2. Experimental Section

This study was reviewed and classified as full committee review research by the Institutional Review Board (IRB) at the University of California, San Diego (UCSD) (Project #121429). The University of California, Irvine (UCI) relied on the IRB approval granted by UCSD (HS #2013-9341).

### 2.1. Participants

All study participants were pregnant women between the ages of 18–50 from San Diego County. Participants in this study had been referred to the Fetal Care Center at the University of California, San Diego Thornton Hospital for genetic counseling after they screened positive for Trisomy 18 or 21 through the CPSP. The majority of the patients seen for genetic counseling at the UCSD Fetal Care Center are high-risk pregnancy patients, including women who screen positive through the California prenatal screen for Trisomies 18 or 21, NTD or SLOS.

Approximately 1200 high-risk pregnancy patients are seen semi-annually for genetic counseling at the UCSD Fetal Care Center. Of these, approximately 250 are referred for a positive Trisomy 18 or 21 California Prenatal Screen result.

Participants were excluded if they were younger than 18 years, had twin pregnancies, or did not meet the referral criterion.

### 2.2. Data Collected

A chart review was performed on each of the participants in this study. Patient information consisting of referral intake information, consultation summary letters, and fetal ultrasound reports was accessed using the UCSD electronic medical record system (EPIC™). Data collected from charts include: visit date, ethnicity, health insurance, number of pregnancies including live births, miscarriages and pregnancy terminations, gestational trimester at positive screen, whether cff-DNA screening was requested or offered, and any additional tests pursued.

Data was collected for two distinct time intervals—A 6-month interval from January to June 2011, before cell-free fetal DNA screening became available, and a 7-month interval from June to December 2012, after cell-free fetal DNA screening was available to all UCSD high-risk prenatal patients.

### 2.3. Cell Free Fetal DNA Screening Laboratory

Cell-free fetal DNA screening was offered solely through Sequenom Laboratories, Inc. (San Diego, CA, USA). The test is specifically known as MaterniT21 Plus and screens for Trisomies 13, 18, and 21. The laboratory reports a turn-around time of 7 business days from receipt of the sample. In our experience, the turn-around time for result reporting was typically 7–8 calendar days. Per a validation study performed, the non-reportable rate for Sequenom Laboratories, Inc. is 0.9% [30]. During the study period, about 4% of patients pursuing cell-free fetal DNA screening were required to have a second sample drawn due to reasons such as low fetal fraction; almost all patients had a successful result after a second specimen was analyzed.

### 2.4. Genetic Counseling Session

During a prenatal genetic counseling session, patients were counseled about the increased risk associated with Trisomy 18 or Trisomy 21 based on their CPSP results. Patients were educated about the risk for intellectual disability, congenital defects, additional future health concerns, and mortality rates associated with each syndrome. In addition, the limitations of screening tests were discussed; specifically focusing on the accuracy of screen tests not being as definitive as invasive tests as they do not look directly at fetal chromosomes. It was emphasized that patients should think about what a confirmatory positive result would mean for them. Patients and genetic counselors would discuss whether a positive result would help them decide on the outcome of the pregnancy. The genetic counseling sessions were a very open and patient guided discussion.

### 2.5. Statistical Methods

Data were compared between the two time periods (before and after cell-free fetal DNA screening). Categorical variables including ethnicity, gestational trimester at positive screen, type of health insurance, type of CPSP screen, referral reason, and the presence of abnormalities seen on the fetal ultrasound were analyzed using Pearson chi-square tests with a two-sided significance level of 0.05. Continuous variables including age, gestational age, and the positive screen value for Trisomy 18 or 21 were compared using a two-group *t*-test. With the available sample size (250 per group), there is 81% power to detect a difference between proportions of 0.13 (odds ratio of 1.7).

## 3. Results

A total of five hundred participants were evaluated in this study: 250 participants in 2011 (before cell-free fetal DNA screening was offered) and 250 participants in 2012 (after cell-free fetal DNA screening was offered). Table 2 summarizes descriptive characteristics of each cohort. Participants in the 2011 and 2012 cohorts averaged 35.1 and 35.2 years of age, respectively. Cohorts did not differ with respect to ethnicity, gestational trimester at positive screen, health insurance type, CPSP screening test, or reason for referral.

### 3.1. Cell-Free Fetal DNA Screening Impact on Testing Chosen

The percentage of participants who chose not to pursue further testing after a positive screen decreased significantly between 2011 and 2012, from 44% (110/250) to 32% (79/250) (*p* = 0.006, Table 3). Of those participants who chose to pursue additional testing in 2011, 47% (117/250) chose invasive testing (either CVS or amniocentesis) and only 29% chose invasive testing in 2012 (*p* < 0.001, Table 3).

**Table 2:** Cohort group statistics.

	Before Cell Free Fetal DNA Screening Was Offered (2011)			After Cell Free Fetal DNA Screening Was Offered (2012)			
	*N*	Mean	SD	*N*	Mean	SD	*t*-Test *p*-Value
**Age**	250	35.06	5.83	250	35.19	5.45	0.80
**Gestational Age**	242	17.37	2.54	240	17.66	2.45	0.20
**Positive Screen Risk for T21**	243	3.88	7.02	243	3.40	5.43	0.40
**Positive Screen Risk for T18**	13	10.75	11.37	11	6.42	9.70	0.33
	***N***	**%**		***N***	**%**		**Chi-Square** ***p*-Value**
**Ethnicity**	250	100.0		250	100.0		0.24
Caucasian, Non-Hispanic	100	40.0		86	34.4		
Hispanic	104	41.6		97	38.8		
Asian	37	14.8		51	20.4		
African American	5	2.0		8	3.2		
Other	4	1.6		8	3.2		
**Gestational Trimester at Positive Screen**	250	100		250	100.0		0.75
First Trimester	55	22		58	23.2		
Second Trimester	195	78		192	76.8		
**Health Insurance**	250	100.0		250	100.0		0.51
HMO	55	22.0		42	16.8		
Medi-Cal	83	33.2		89	35.6		
PPO	100	40.0		108	43.2		
**CPSP Screening Test**	250	100.0		250	100.0		0.85
First Trimester Combined	55	22.0		59	23.6		
Quadruple *	66	26.4		67	26.8		
Serum Integrated	60	24.0		52	20.8		
Sequential Integrated	69	27.6		72	28.8		
**Referral Reason**	250	100.0		250	100.0		0.60
Positive Trisomy 18 Screen	7	2.8		7	2.8		
Positive Trisomy 21 Screen	237	94.8		240	96.0		
Positive T21 & T18 Screen	6	2.4		3	1.2		

Standard Deviation (SD), Trisomy 21 (T21), Trisomy 18 (T18), Health Maintenance Organization (HMO), Preferred Provider Organization (PPO), California Prenatal Screening Program (CPSP), Nuchal Translucency (NT). * The Quadruple and Quadruple + NT screen categories were combined for the Pearson chi-squared test because of the small number of participants in the Quadruple + NT category.

**Table 3:** Cell-free fetal DNA screening impact on testing chosen by year and trimester.

	Before Cell-Free Screening Offered (2011)		After Cell-Free Screening Offered (2012)		Chi-square Test (2011 *vs.* 2012)
	*N*	%	*N*	%	*p*-Value
**Testing Chosen**	**250**	**100.0**	**250**	**100.0**	
**No further testing**	**110**	**44.0**	**79**	**31.6**	**0.006 ^a^**
**Invasive testing**	**117**	**46.8**	**72**	**28.8**	**<0.001 ^b^**
CVS	4		2		
Amniocentesis	113		70		
**Non-invasive testing**	**23**	**9.2**	**99**	**39.6**	
Sequential Integrated	23		24		
Cff-DNA Screening	NA		75		
**First Trimester test**	**55**	**100.0**	**58**	**100.0**	
**No further testing**	**3**	**5.5**	**2**	**3.5**	**0.674 ^c^**
**Invasive testing**	**29**	**52.7**	**17**	**29.3**	**0.008 ^b^**
CVS	4		2		
Amniocentesis	25		15		
**Non-invasive testing**	**23**	**41.8**	**39**	**67.2**	
Sequential Integrated	23		24		
cff-DNA Screening	NA		15		
**Second Trimester test**	**195**	**100.0**	**192**	**100.0**	
**No further testing**	**107**	**54.9**	**77**	**40.1**	**0.004 ^a^**
**Invasive testing**	**88**	**45.1**	**55**	**28.6**	0.004 ^b^
CVS	NA		NA		
Amniocentesis	88		55		
**Non-invasive testing**	**NA**		**60**	**31.3**	
Sequential Integrated	NA		NA		
cff-DNA Screening	NA		60		

NA: Test not available. ^a^ *Χ*^2^ (df = 1) test for difference in % who chose additional testing *vs.* no further testing; ^b^ *Χ*^2^ (df = 1) test for difference in % who chose invasive testing *vs.* non-invasive testing; ^c^ Fisher’s Exact test for difference in % who chose additional testing *vs.* no further testing.

### 3.2. Cell-Free Fetal DNA Screening Impact on Testing Chosen by Trimester

Of the participants who received a positive screen result in the first trimester, the majority of individuals who screened positive in the first trimester chose to pursue additional testing, specifically CVS, amniocentesis, sequential integrated screening, or, in 2012, cell-free fetal DNA screening (Table 3). There was no significant change from 2011 to 2012 in the percent who chose no further testing among participants who screened positive in the first trimester (*p* = 0.674). However, individuals chose significantly fewer invasive tests compared to non-invasive tests in 2012—from 53% (29/55) in 2011 to 29% (17/58) in 2012 (*p* = 0.008).

Participants who screened positive in the second trimester did not have the option of pursuing additional serum screening through the CPSP. Additional testing for these women included only invasive testing in 2011 and the addition of cell-free fetal DNA screening in 2012. There was a significant decrease in the percentage who chose no further testing—from 55% (107/195) in 2011 to 40% (77/192) in 2012 (*p* = 0.004, Table 3). In addition, participants chose significantly fewer invasive tests compared to non-invasive tests—from 45% (88/195) in 2011 to 29% (55/192) in 2012 (*p* = 0.004).

### 3.3. Cell-Free Fetal DNA Screening Impact on Testing Chosen by Health Insurance

Insurance type influenced whether or not further testing was pursued in both 2011 and 2012 (Table 4). In each year, a significantly higher proportion of PPO and HMO patients chose additional testing compared to Medi-Cal (California’s Medical Assistance Medicaid Program) or unknown insurance (*p* = 0.046 for 2011, *p* < 0.001 for 2012). The likelihood of no further testing decreased significantly from 37% in 2011 to 16% in 2012 for participants who carried PPO insurance (*p* = 0.009). Among patients with HMO insurance, a non-significant decline was observed from 2011 to 2012 in the percentage choosing no further testing (38% to 30%, *p* = 0.133). However, no difference was observed among patients with Medi-Cal insurance (53% in 2011 *vs.* 50% in 2012, *p* = 0.75). The percentage choosing invasive testing *vs.* non-invasive testing decreased significantly from 2011 to 2012 for each type of health insurance (Table 4). The frequency of invasive testing decreased by nearly 50% for PPO, HMO and unknown insurance, while the decline was smaller for those with Medi-Cal insurance.

**Table 4:** Cell-free fetal DNA screening impact on testing chosen by health insurance.

	Before Cell-Free Screening Offered (2011)		After Cell-Free Screening Offered (2012)		Chi-square Test (2011 *vs.* 2012)
	*N*	%	*N*	%	*p*-Value
**Health Insurance—Medi-Cal**	**83**	**100.0**	**89**	**100.0**	
**No further testing**	**44**	**53.0**	**45**	**50.6**	**0.748 ^a^**
**Invasive testing**	**32**	**38.6**	**27**	**30.3**	**0.038 ^b^**
CVS	0		0		
Amniocentesis	32		27		
**Non-invasive testing**	**7**	**8.4**	**17**	**19.1**	
Sequential Integrated	7		3		
cff-DNA Screening	NA		14		
**Health Insurance—PPO**	**100**	**100.0**	**108**	**100.0**	
**No further testing**	**37**	**37.0**	**18**	**16.7**	**0.009 ^a^**
**Invasive testing**	**53**	**53.0**	**32**	**29.6**	**<0.001 ^b^**
CVS	3		2		
Amniocentesis	50		30		
**Non-invasive testing**	**10**	**10.0**	**58**	**53.7**	
Sequential Integrated	10		15		
cff-DNA Screening	NA		43		
**Health Insurance—HMO**	**55**	**100.0**	**42**	**100.0**	
**No further testing**	**21**	**38.2**	**10**	**23.8**	**0.133 ^a^**
**Invasive testing**	**28**	**50.9**	**11**	**26.2**	**<0.001 ^b^**
CVS	1		0		
Amniocentesis	27		11		
**Non-invasive testing**	**6**	**10.9**	**21**	**50.0**	
Sequential Integrated	6		6		
cff-DNA Screening	NA		15		
**Health Insurance—Unknown**	**12**	**100.0**	**11**	**100.0**	
**No further testing**	**8**	**66.7**	**6**	**54.5**	**0.529 ^a^**
**Invasive testing**	**4**	**33.3**	**2**	**18.2**	**0.167 ^c^**
CVS	0		0		
Amniocentesis	4		2		
**Non-invasive testing**	**0**	**0.00**	**3**	**27.3**	
Sequential Integrated	0		0		
cff-DNA Screening	NA		3		
*Χ*^2^ (df = 3) test for difference in % who chose additional testing *vs.* no further testing by health insurance type	***p* = 0.046**		***p* < 0.001**		

NA: Test not available. ^a^ *Χ*^2^ (df = 1) tests difference in % who chose additional testing *vs.* no further testing; ^b^ *Χ*^2^ (df = 1) tests difference in % who chose invasive testing *vs.* non-invasive testing; ^c^ Fisher’s Exact test for difference in % who chose invasive testing *vs.* non-invasive testing.

### 3.4. Cell-Free Fetal DNA Screening Impact on Testing Chosen by Presence of Ultrasound Abnormalities

The presence of fetal ultrasound abnormalities increases the likelihood of chromosomal aneuploidy, such as Trisomies 13, 18, and 21. Ultrasound abnormalities were identified in 53/201 and 52/199 patients who qualified for a detailed fetal anatomy scan (ultrasound) at the time of their positive screen in 2011 and 2012, respectively (Table 5). A non-significant increase in the percentage who chose no testing from 36% in 2011 to 44% in 2012 was seen (*p* = 0.381). However, among participants without ultrasound abnormalities, the percentage who chose no further testing decreased significantly from 60% (88/148) in 2011 to 37% (55/147) in 2012 (*p* < 0.001). Additionally, in 2011 a smaller percentage of participants with ultrasound abnormality (36%) chose no further testing compared to those without ultrasound abnormality (60%) (*p* = 0.003). However, in 2012, after the introduction of cell-free fetal DNA screening, the percentage of participants with ultrasound abnormality and without ultrasound abnormality who chose no further testing (44% and 37%, respectively) were not significantly different (*p* = 0.387). In addition, participants who had ultrasound abnormalities identified in the fetus chose invasive testing less often after cell-free fetal DNA screening was introduced (*p* = 0.002). A parallel trend was also identified for participants without ultrasound abnormalities (*p* < 0.001).

**Table 5:** Cell-free fetal DNA screening impact on testing chosen by ultrasound abnormalities.

	Before Cell-Free Screening Offered (2011)		After Cell-Free Screening Offered (2012)		Chi-square Test (2011 *vs.* 2012)
	*N*	%	*N*	%	*p*-Value
**Abnormalities Present on 2nd Trimester Ultrasound**	**53**	**100.0**	**52**	**100.0**	
**No further testing**	**19**	**35.8**	**23**	**44.2**	**0.381 ^a^**
**Invasive testing**	**32**	**60.4**	**17**	**32.7**	**0.002 ^c^**
CVS	NA		NA		
Amniocentesis	32		17		
**Non-invasive testing**	**2**	**3.8**	**12**	**23.1**	
Sequential Integrated	2		0		
cff-DNA Screening	NA		12		
**No Abnormalities Present on 2nd Trimester Ultrasound**	**148**	**100.0**	**147**	**100.0**	
**No further testing**	**88**	**59.5**	**55**	**37.4**	**<0.001 ^a^**
**Invasive testing**	**60**	**40.5**	**41**	**27.9**	
CVS	NA		NA		
Amniocentesis	0		41		
**Non-invasive testing**	**0**	**0.00**	**51**	**34.7**	**<0.001 ^c^**
Sequential Integrated	0		2		
cff-DNA Screening	NA		49		
*Χ*^2^ (df = 1) test for difference in % who chose additional testing *vs.* no further testing by presence of U.S. abnormality	***p* = 0.003**		***p* = 0.387**		

NA: Test not available. ^a^ *Χ*^2^ (df = 1) tests difference in % who chose additional testing *vs.* no further testing; ^c^ Fisher’s Exact test for difference in % who chose invasive testing *vs.* non-invasive testing.

## 4. Discussion

The results of this study showed an increase in the number of participants who chose to pursue follow-up testing after a positive screen in 2012 compared to 2011. However, approximately one-third of women chose not to pursue additional testing. There are several factors that could have been responsible for this decision, including trimester at which a woman screened positive, financial concerns, and the decision to not to pursue testing because it would not change the outcome of the pregnancy.

This study alludes to the impact that cell-free fetal DNA screening has had on prenatal patients. The trends identified in this study have been seen in the past after the introduction of new prenatal screening tests. For example, there was a significant decrease in the demand for invasive testing after the introduction of NT measurements [32,33] A decrease in the number of invasive procedures was also seen after first trimester combined screening was made available to Medi-Cal (state insured) patients [34]. This suggests that when cell-free fetal DNA screening becomes validated in lower-risk populations, it will continue to exceed other screening methods in popularity and further reduce the number of invasive tests performed. However, because the false positive rate still exists, albeit low, invasive testing is recommended to confirm a positive screen result. This leads to the concern that if cell-free fetal DNA screening is performed later in the pregnancy, the results may be available too late to pursue additional, invasive testing.

There are several possible reasons why women who screened positive during the first trimester chose invasive testing less often after the introduction of cell-free fetal DNA screening. First, since women could have a highly accurate screening test earlier in the pregnancy and still have the option to choose an invasive procedure during the second trimester, they may have felt more secure in choosing the screening test. Second, women who screened positive toward the end of the first trimester may have been too late in gestation to have a CVS but too early to have an amniocentesis and may have chosen a non-invasive screening test in the intervening time. Third, the availability of cell-free fetal DNA screening at 10 weeks gestation may makes it possible for women to get answers earlier in the pregnancy rather than waiting until the second trimester. A study showed a strong preference for first trimester screening when women were offered both first trimester and second trimester prenatal screening [35]. Another study showed that women prefer first trimester screening because it gives them reassurance earlier in the pregnancy [36].

Woman who screened positive during the second trimester of pregnancy chose to pursue additional testing more often and chose invasive testing less often after the introduction of cell-free fetal DNA screening. There are many factors that could be responsible for this difference. Women who screened positive during the second trimester may have decided that additional testing at a later stage in the pregnancy would not change their decision regarding whether to terminate or to continue the pregnancy if a chromosomal aneuploidy were to be identified. Therefore, they may not have felt the need to pursue additional testing. In addition, women who screened positive on a Quadruple screen, with only one blood draw during the second trimester, may have begun their prenatal care later in the pregnancy and were too late in gestation to pursue invasive testing. Delayed prenatal care often occurs with lower socioeconomic background. However, because data on socioeconomic status were not collected for this study, it was not possible to test this hypothesis.

Type of insurance was also associated with the choice to pursue cell-free fetal DNA screening. Participants who carried PPO insurance chose to pursue additional testing more often after cell-free fetal DNA screening became available. This trend was not observed in participants with Medi-Cal insurance. Another interesting finding was that patients with Medi-Cal insurance chose additional testing less frequently in both years than patients with HMO or PPO insurance. In addition, participants with Medi-Cal insurance chose invasive testing with nearly the same frequency over the two-year period, whereas there was a sharp decline in invasive testing for those with PPO or HMO insurance. Patients with Medi-Cal insurance typically have lower income and socioeconomic status. The added cost and/or time required for cell-free fetal DNA screening may not have been acceptable for them. Cost may be a major factor behind these trends. At UCSD, patients whose health insurance did not cover cff-DNA screening were responsible for varying proportions of cost for the test. A patient with a PPO was responsible for a maximum of $235 while a patient with HMO insurance was responsible for a maximum of $475. A patient with Medi-Cal insurance could be responsible for up to the full cost of the test, $1,900, depending on her income. The high cost of testing for HMO or Medi-Cal carriers could have been a strong factor in dissuading them from pursuing additional testing. A 2006 study by Hall, *et al.* showed that participants were more likely to choose genetic testing that was “low cost [37]”.

Participants with ultrasound abnormalities identified in the pregnancy did not choose additional testing more often after the introduction of cell-free fetal DNA screening. They did, however, choose invasive testing less often. This is an interesting trend to see as abnormal ultrasound findings can increase the likelihood of chromosomal aneuploidy in the fetus [38] and the definitive test to diagnose chromosomal aneuploidy is an invasive test. It is difficult to identify a cause for this trend; however, it can be postulated that this trend may be seen due to the gestational trimester at which these abnormalities are detected. Women may choose not to do additional testing because they felt their decision to continue the pregnancy would not be altered by additional information.

Since this study was a retrospective chart review, it is difficult to identify other changes over time besides the introduction of cell-free fetal DNA testing that could have impacted choices made by patients. There is also the possibility that the data in the electronic medical records were inaccurate or incomplete. The dates for the study were chosen to reduce this limitation, since most of the patients had already had their final clinic visit by the time their chart reviews were performed. In addition, this study was unable to capture the effect that a positive cell-free fetal DNA screen result had on the number of affected pregnancy terminations. Data were not collected on pregnancy outcomes after additional follow-up testing identified an affected fetus. 

## 5. Conclusions

Cell-free fetal DNA screening has impacted the testing choices for women who have screened positive through the CPSP. The rate of additional testing increased, and the rate of invasive testing decreased.
